# Supplementary material for: A rock-boring and rock-ingesting freshwater bivalve (shipworm) from the Philippines
Source: Proc Biol Sci. 2019 Jun 19;286(1905):20190434. doi: 10.1098/rspb.2019.0434 (PMC6599978; doi:10.1098/rspb.2019.0434)

**Supplemental Table 1: Diagnostic characters of teredinid genera closely related to *Lithoredo*.**

| **Taxonomic Character** | ***Lithoredo*** | ***Teredora*** | ***Uperotus*** | ***Tamilokus*** |
| --- | --- | --- | --- | --- |
| Labial Palps | Large & free | Free | Large & free | Reduced/absent |
| Gills | Extend from base of siphons to mouth | Extend from base of siphons to mouth | Extend from base of siphons to mouth | Located posteriorly, extending to posterior caecum |
| Crystalline Style | Located anterior to posterior adductor muscle | Located anterior to posterior adductor muscle | Located anterior to posterior adductor muscle | Extends from base of foot beyond posterior adductor muscle |
| Stomach | Globular, located anterior to posterior adductor muscle | Globular, located anterior to posterior adductor muscle | Globular, located anterior to posterior adductor muscle | Globular, located posterior to posterior adductor muscle |
| Caecum | Absent | Doubles back upon itself, to right | Doubles back upon itself, to right | Doubles back upon itself, to right |
| Intestine | Extends medially into anal canal | Extends anteriorly into anal canal | Extends anteriorly into anal canal | Extends anteriorly into anal canal |
| Heart | Median position | Anterior, located dorsal to caeca | Anterior, located dorsal to caeca | Median position |
| Siphons | United 3/4 length, separate at tip | United to tip | United to tip | Separated along entire length, pink pin-striped pigmentation |
| Incurrent Siphon Papillae | 1st/2nd row small, 3rd inner row large papillae, brown pigmentation at tip | Incurrent siphon numerous large papillae | Incurrent siphon numerous small papillae | Primary row numerous small papillae, secondary row compound papillae |
| Excurrent Siphon Papillae | Triple-row small papillae, brown pigmentation at tip | - | Excurrent siphon two large papillae on dorsal surface | Numerous small papillae |
| Cephalic Hood | Inconspicuous, does not cover valve posterior slope | Inconspicuous, does not cover valve posterior slope | Inconspicuous, does not cover valve posterior slope | Prominent, covers valve posterior slope |
| Cephalic Collar | Absent | Absent | Absent | Present |
| Pallets | Thick, calcareous, OF deep 'thumbnail' depression, IF medially-divided | Paddle shaped, non-segmented, 'thumbnail' depression | Paddle shaped, non-segmented, 'thumbnail' depression, radiating ribs | Triangular, cup-shaped, non-segmented, ovate flattened stalk |
| Valve Denticles | Denticulated structures large, broad, blunt and spatulate | Denticulated structures fine and sharp | Denticulated structures fine and sharp | Denticulated structures fine and sharp |
| Mantle | Thick, white, opaque | Thin, white-translucent | Thin, white-translucent | Thin, white-translucent |

**Supplemental Figure 1: Nestling macroinvertebrates associated with the burrows of *Lithoredo abatanica*.** Macroinvertebrates in empty burrow galleries, including crabs, shrimp, limpets, gastropods and clams. Scale bar = 10 mm.


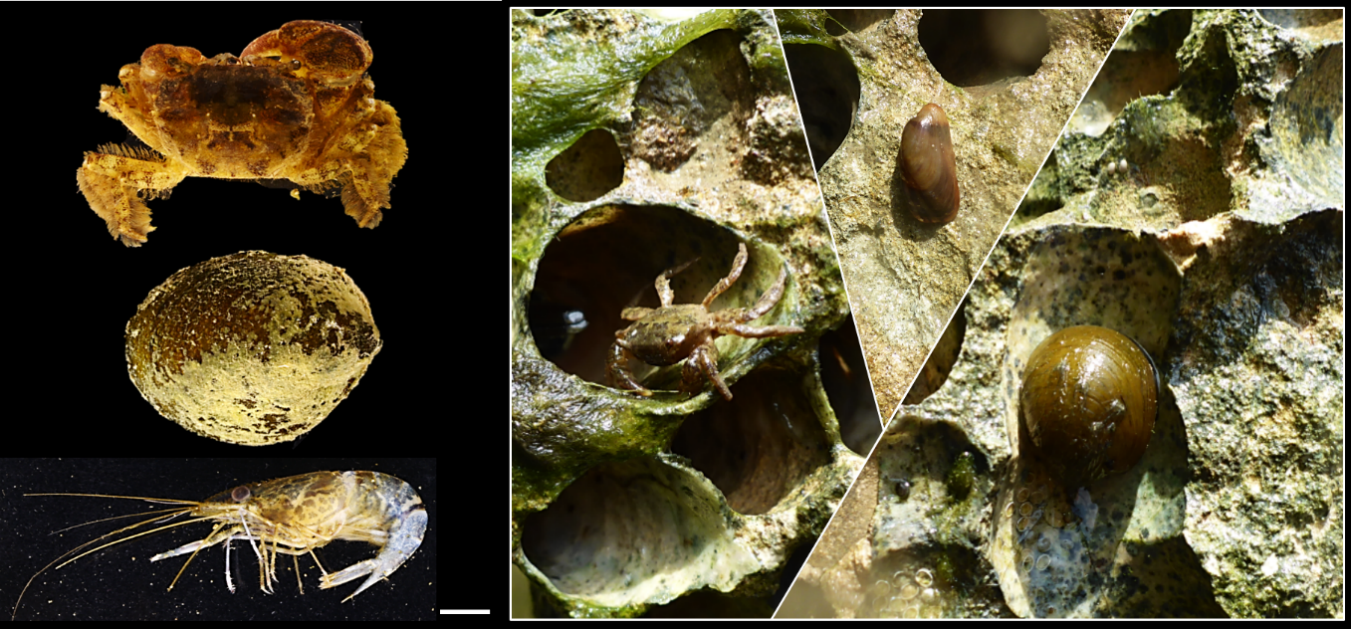


**Supplemental Figure 2. Supplemental Figure 2: Calcareous shell valves of *Lithoredo abatanica*:** A, MicroCT 3D render of dorsal animal (specimen PMS-4314K); B, outer face shell valves (specimen PMS-4130P); C, inner face shell valves (specimen PMS-4130P). D and E, inner and outer surface of shell valves and pallets across ontogeny. A, apophysis; AS, anterior slope; DC, dorsal condyle; MS, median slope; PAM, posterior adductor muscle; PB, pallet blade; PS, posterior slope; Si, siphon; SV, shell valve; VC, ventral condyle. Scale bar for A, B-C and D-E = 2.5 mm, 1 mm and 2 mm respectively.


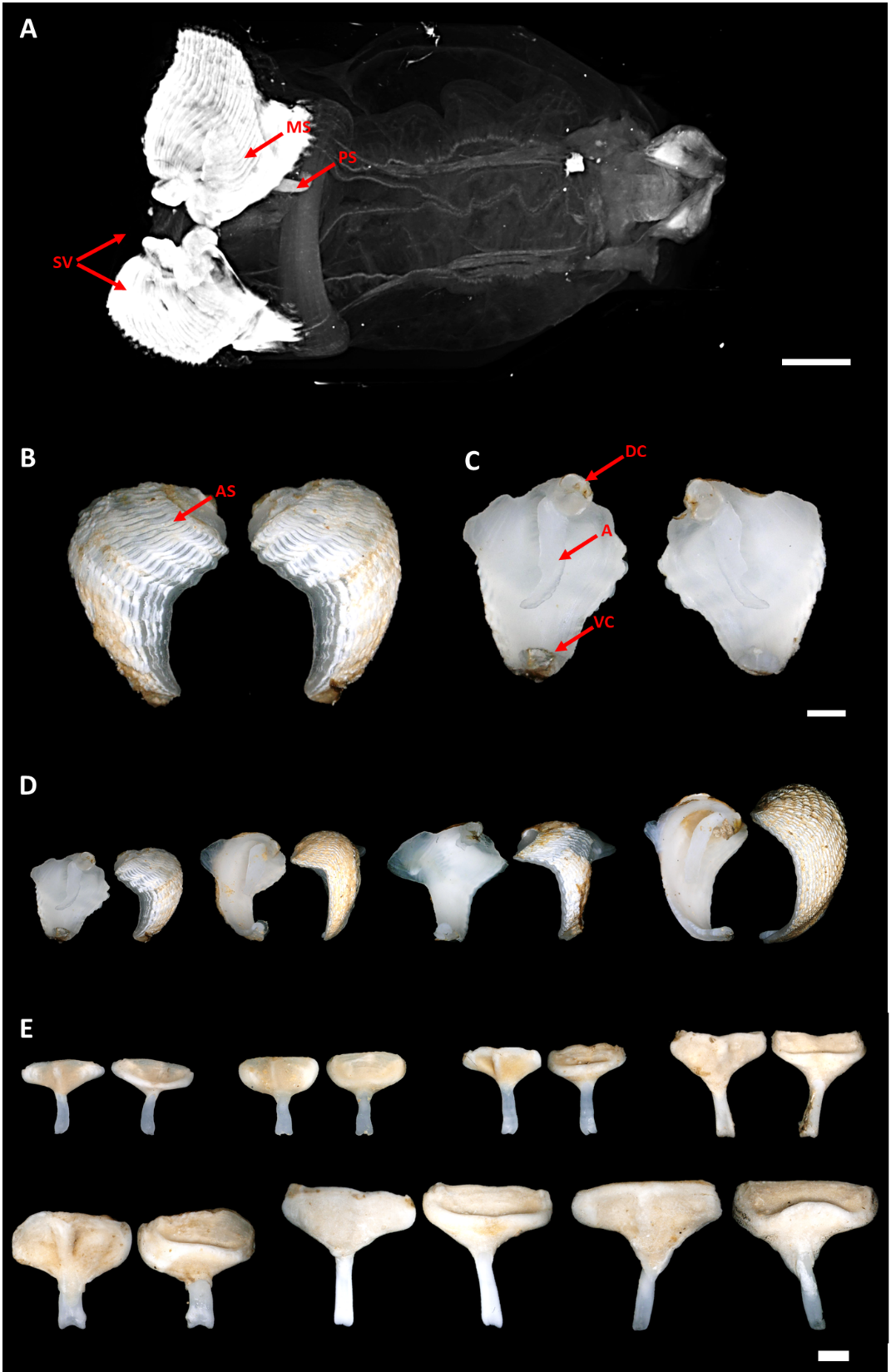


**Supplemental Figure 3: A 3D digital MicroCT render of *Lithoredo abatanica*.**


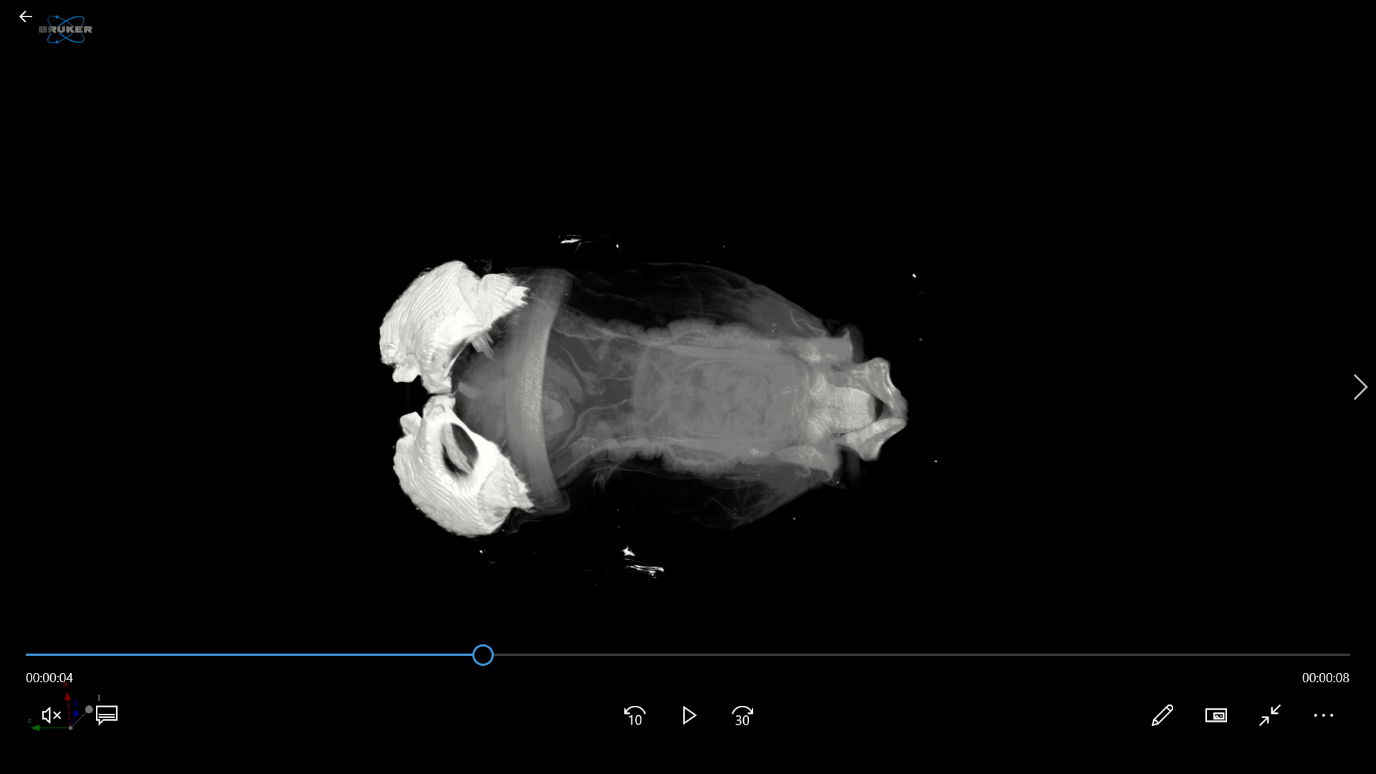


**Supplemental Figure 4: Phylogenetic position of *Lithoredo abatanica* within Teredinidae.** A full tree from a Bayesian analysis of the concatenated 18S and 28S nuclear rRNA gene sequences obtained from specimen PMS-4316M. Numbers at nodes indicate posterior probabilities. Scale bars denote nucleotide substitutions per site.

**Supplemental Figure 5: Symbiotic bacteria associated with the gill of *Lithoredo abatanica*.** A, scanning electron micrograph depicting bacteria exposed by fracturing the gill along the plane perpendicular to its long axis; B, boxed region from A. Scale bars for A-B = 100 µm and 20 µm respectively.


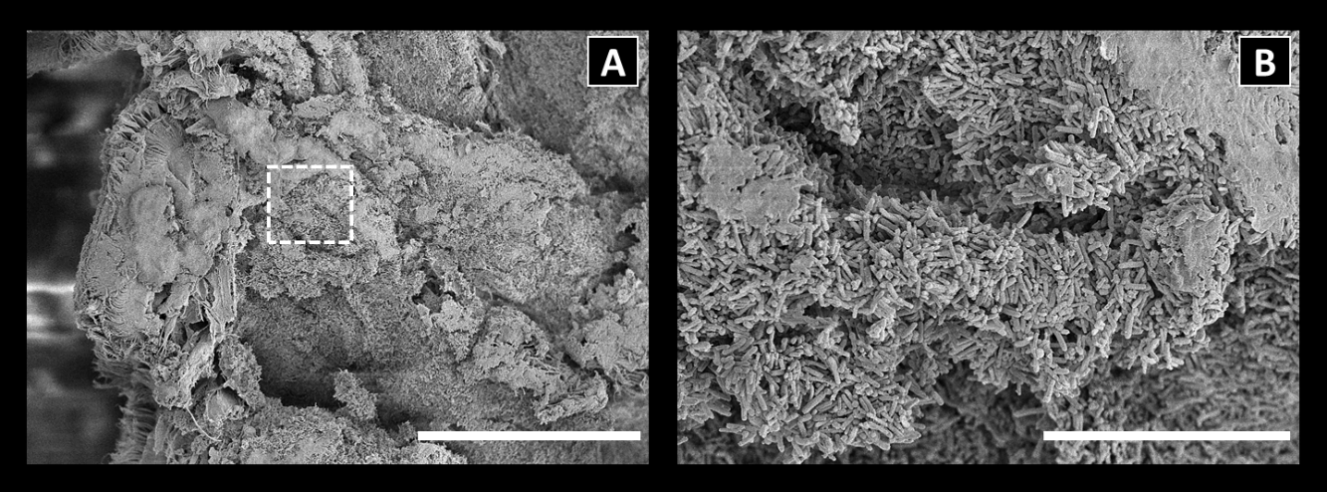


**Supplemental Figure 6: Anatomy and mineralogical analyses of the gut content of *Lithoredo abatanica*:** A, Scanning electron micrograph of a transverse section through the intestine and gonad; B, boxed region from A showing detail of the gut and gonad; C, energy dispersive X-ray (EDX) analysis demonstrates that the elemental composition of intestinal content and rock substrate are nearly identical (averaged across three individuals), while that of the gonad tissue differs markedly; D, X-ray diffraction (XRD) mineralogical analysis shows near identical spectra generated by intestinal content rock substrate and a calcite reference standard. CT, carbon tab (negative control); Go, gonad; IC, intestinal content; IE, intestinal epithelium. Scale bar = 100 µm and 25 µm respectively.


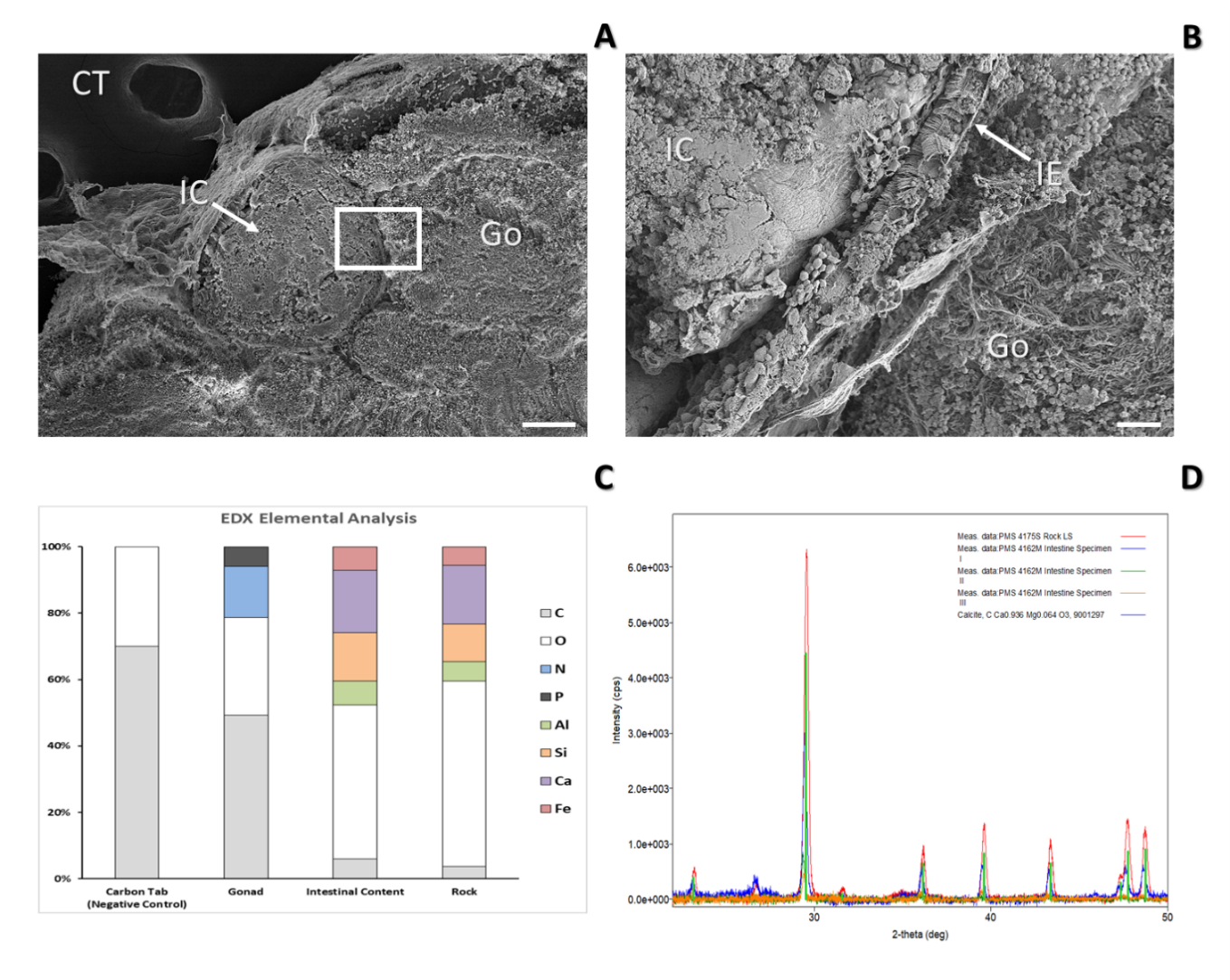

Supplement: Supplemental Table and Figures [file rspb20190434supp1.docx]
